# An embedded gene selection method using knockoffs optimizing neural network

**DOI:** 10.1186/s12859-020-03717-w

**Published:** 2020-09-22

**Authors:** Juncheng Guo, Min Jin, Yuanyuan Chen, Jianxiao Liu

**Affiliations:** 1 Hubei Key Laboratory of Agricultural Bioinformatics, College of Informatics, Huazhong Agricultural University, Wuhan, 430070 China; 2 Institute of Information Engineering, Chinese Academy of Sciences, Beijing, 10049 China; 3 School of Cyber Security, University of Chinese Academy of Sciences, Beijing, 10049 China; 4 National Key Laboratory of Crop Genetic Improvement, Huazhong Agricultural University, Wuhan, 430070 China

**Keywords:** Gene mining, Neural network, Knockoffs, Nonlinear data, Maize

## Abstract

**Background:**

Gene selection refers to find a small subset of discriminant genes from the gene expression profiles. How to select genes that affect specific phenotypic traits effectively is an important research work in the field of biology. The neural network has better fitting ability when dealing with nonlinear data, and it can capture features automatically and flexibly. In this work, we propose an embedded gene selection method using neural network. The important genes can be obtained by calculating the weight coefficient after the training is completed. In order to solve the problem of black box of neural network and further make the training results interpretable in neural network, we use the idea of knockoffs to construct the knockoff feature genes of the original feature genes. This method not only make each feature gene to compete with each other, but also make each feature gene compete with its knockoff feature gene. This approach can help to select the key genes that affect the decision-making of neural networks.

**Results:**

We use maize carotenoids, tocopherol methyltransferase, raffinose family oligosaccharides and human breast cancer dataset to do verification and analysis.

**Conclusions:**

The experiment results demonstrate that the knockoffs optimizing neural network method has better detection effect than the other existing algorithms, and specially for processing the nonlinear gene expression and phenotype data.

## Introduction

In recent years, large amounts of biological data (such as genomes, transcriptomes, and phenotypes) have been generated with the maturity and rapid development of many high-throughput technologies. In this context, it’s possible to mine gene loci for specific phenotypic traits (such as crop vitamin A content, agronomic traits, human diseases, *etc*) from the genome-wide data. In recent years, Genome-Wide Association Study (GWAS) and linkage analysis have become important ways of gene location and fine allele discovery. At present, a lot of quantitative trait loci controlling various phenotypic traits have been mapped by biologists using these methods. However, linkage analysis method needs to construct segregated population, longer cycle and low accuracy. Generally, it can only limit the Quantitative Trait Locus (QTL) within 10 cM and 20 cM, and the work of QTL fine mapping is time-consuming and labor-consuming. At the same time, the statistical efficacy of GWAS is relatively low. Generally, this method can only locate the major QTL, and the false positive rate is relatively high.

Gene selection refers to find a small subset of discriminant genes for specific phenotypic traits using microarray data. Gene selection plays an important role in gene expression analysis, and it has important research significance in increasing crop yields, improving crop quality, diagnosing and treating human diseases. The bioinformatics-based gene selection method overcomes the shortcomings of traditional biological experiment methods, such as high cost, time-consuming and laborious, etc. The bioinformatics-based methods mainly use machine learning related algorithms to perform biological computation, and thus to mine pathogenic genes. At present, it has become a research hotspot of bioinformatics and an effective method of pathogenic gene mining. The main challenge of selecting genes is that there are fewer samples, more features (genes), and higher noise in the data. At present, there are mainly three kinds of machine learning related gene selection methods: filter method, wrapper method and embedded method.

1. The filter method mainly refers to score each feature gene according to divergence or correlation, and select genes by setting a threshold. The often used filter method includes 푡-test feature selection [1], correlation-based feature selection (CFS) [2], information gain [3], chi-square test, mutual information [4], etc. The filter method is easy to implement, but it ignores the complex interactions between genes. Therefore, the gene selection accuracy of filter method is often worse than other kinds of methods.
2. The wrapper method mainly uses some intelligent optimization algorithms to search the optimal genes in the feature space. For example, genetic algorithm finds the smaller set of feature genes that the optimization criterion does not deteriorate [5, 6]. The ant colony optimization [7] and artificial bee colony (ABC) algorithms [8] are also used into gene selection. Some research work combines the intelligent optimization algorithms with other methods to realize gene selection. The representative methods include using information gain and swarm optimization algorithm [9], support vector machine (SVM) and artificial bee colony [10], cellular learning automata and ant colony algorithm [11], evolutionary and artificial intelligence method [12], genetic operators [13], etc. In recursive feature eliminating method, it eliminates some features after each training and carries out next training based on the new feature set until satisfying the requirements [14]. This method can obtain the best performance through optimizing the objective function, but the computational complexity is often too large. In addition, Markov blanket [15] and sequential search-based algorithm [16] are also used to select important genes for specific phenotypes.
3. The embedded method takes feature gene selection as a part of the model building. It trains data using some machine learning algorithms and obtains the weight coefficients of each feature gene. Then it selects important genes according to the weight coefficients. The typical embedded methods mainly include random forest [17, 18], regularized logistic regression [19], least absolute shrinkage and selection operator (LASSO) [20], ridge regression [21], elastic net [22] and so on. Among them, random forest method improves the gene selection performance by combining multiple decision trees. It selects the important genes by ranking each feature after training. LASSO constructs a linear model through setting some feature coefficients to zero and uses the nonzero ones as the selected genes.

As we known, the relationship between gene expression and phenotype is often complex and nonlinear. Through using multiple hidden layers and activation functions, neural network has the characteristics of better fitting ability in dealing with complex non-linear data, automatic feature extraction and good flexibility. This work includes the following three aspects:

1. We propose an embedded method, which integrates gene selection into the process of neural network training. After the training is completed, we can evaluate the importance of genes according to the calculated weight coefficient of each gene.
2. Neural networks are often considered as a black-box model due to its internal complexity. It is difficult to discover the key genes that affect the decision-making of neural networks. In order to make the training results interpretable, we introduce the idea of knockoffs into the neural network [23]. By constructing the knockoff feature genes of the original feature genes [24], this method not only make each feature gene to compete with each other, but also make each feature gene compete with its knockoff feature gene. That is to say, we can evaluate the importance of genes for the phenotype traits with the help of the knockoff feature genes.
3. We use the real maize carotenoids, tocopherol methyltransferase, raffinose family oligosaccharides and human breast cancer dataset to do evaluation and validation. Experiment results show the knockoffs optimizing neural network method can mine candidate genes more effectively when to deal with complex non-linear data with independently identically distribution. It has better detection effect compared with the existing 5 kinds of commonly used gene selection methods.

## Results

### Dataset

We have assembled a global maize germplasm collection with 527 inbreds for association mapping panel (*AMP*) with different populations (including 143 lines for *NSS*, non-stiff-stock; 33 for *SS*, Stiff-stock; 232 for *TST*, Tropical and Semi-tropical; and the left 119 are regarded as *MIXED*). This population is released from the major temperate and tropical/subtropical breeding programs of China, International Maize and Wheat Improvement Center (*CIMMYT*) and the Germplasm Enhancement of Maize (*GEM*) project in the US, which were chosen to be the representative of maize genetic diversity and/or for their promise in maize improvement. All of the lines were previously assayed by the 50 K Maize SNP array (commercially available from Illumina). Deep RNA sequencing was also performed on 368 of the 527 lines using kernels harvested 15 days after pollination (DAP). We get about 1 million 60 thousand high quality SNP markers and expression of 28,769 genes, which cover about 70% of the predicted genes in maize genome [25]. All the dataset can be got through http://www.maizego.org/ and http://modem.hzau.edu.cn/ [26].

To evaluate the effectiveness of our method, we chose 4 published genes loci harboring the well-known genes, that is, *crtRB1* and *lycE* for maize carotenoids pathway [27, 28], *VTE4* for maize tocopherol methyltransferase [29], *ZmGOL* for maize raffinose family oligosaccharides [30], of which the four are well-known for their function variation through expression. We select a region upstream and downstream of the 4 noted genes within 1 MB respectively to do the experiment.

It has been reported that in *Zea mays crtRB1* (also known as *HYD3*) were significantly associated with carotenoid variation in association panel, and alleles associated with reduced transcript expression correlate with higher β-carotene concentrations [27]. *lycE*, which encodes a lycopene beta cyclase, commits the first branch point of lycopene cyclization [31]. Previous studies have shown that the transcriptional regulation of *lycB* and *lycE* are the critical regulatory points in carotenoid biosynthesis [32, 33]. *VTE4*, which encodes the γ-tocopherol methyltransferase, is a major gene involved in natural phenotypic variation of α-tocopherol. The reported natural variation in *ZmVTE4* promoter region among the association panel affect kernel α-tocopherol content through regulation gene expression [34]. *ZmGOL*, galactinol synthase 1, is the gene with function of regulating seed vigor by manipulation of raffinose family oligosaccharides [30].

### Experiment comparison and analysis

We compare the performance of our methods with five baseline methods: random forest (*RF*) [17, 18], support vector regression with linear kernel function (*SVR*_*LKF*) [35], mutual information (*MI*) [36], elastic net [22], neural network without knockoffs(*Non-Knockoffs*). Our knockoffs optimization neural network gene selection method in this work is named as *Knockoffs-NN*. In the method of *RF*, we measure the importance of each feature through calculating the Gini coefficient [18]. In *SVR*_*LKF*, we measure the importance of each feature by the coefficients in the primal problem [35]. In *MI*, we get the relevance between each gene and phenotype trait through calculating the mutual information.

We can get the ranking of target gene according to the weight coefficient has been calculated in different methods. We use Eq. (1) to calculate the power of the target gene. In the equation, *rank* refers to the ranking of the target gene and *num* refers to the total number of genes in the dataset. Obviously, higher ranking of target gene denotes the greater of the corresponding power.

1 $$ power=1-\frac{\mathit{\operatorname{rank}}}{num} $$

In *Knockoffs-NN*, we first load the data to generate a *m* × *n* matrix, which is denoted as *X* ∈ ℝ^*m* × *n*^. It means there are *n* genes and *m* samples in the dataset. The element *x*_*ij*_ in *X* represents the expression of the *j*-th gene in the *i*-th sample. *Y* ∈ ℝ^*m* × 1^ represents the phenotype trait of each sample. We do the standardization on the dataset *X* firstly, and generate knockoff feature genes using MATLAB based on the reference code in [24] (http://web.stanford.edu/group/candes/knockoffs/software/knockoffs/). Then we can get a *m* × 2*n* matrix, in which the first *n* dimension represent the original feature genes and next *n* dimension represent the knockoff feature genes. We take the *m* × 2*n* matrix as the input of our neural network (Fig. 7). Then we get a *m* × *n* matrix after coupling through the coupling layer. The multi-layer perceptron (*MLP*) is used to learn the function from input *n* feature genes to the output *Y*. After the training is completed, we evaluate the importance of each gene through calculating the weight coefficient (Eq. (10)). In order to ensure the accuracy comparison, we train the neural network 10 times with different random seeds and obtain the average weight coefficient of each feature gene. In our experiment, we use *ReLU* activation function, *L1* regularization and mean square error loss function. We use the batch gradient descent method and optimize the loss function by the Adam algorithm. The hyperparameters setting (such as learning rate, number of hidden layers) in our experiments is shown in Table 1.

**Table 1:** Parameters setting in the experiment

Parameter setting	Value
Activation function	ReLU
Regularization	L1
Loss function	Mean square error (MSE)
Optimization	Batch gradient descent and Adam (recommend mini-batch for large samples)
Number of hidden layer	1
Number of hidden layer neurons (genes)	Number of genes
Learning rate	0.0001

### Validation of *crtRB1* for maize carotenoids

**Fig. 1:**
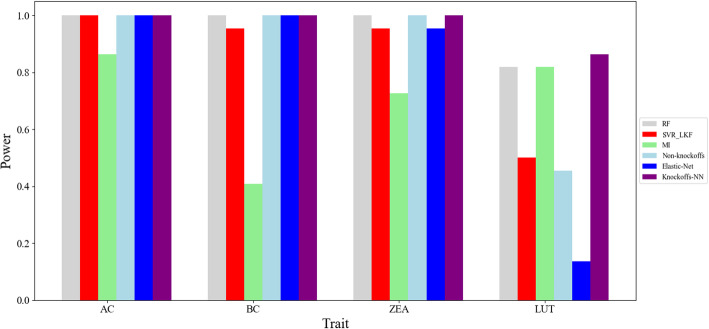
Result of gene *crtRB1*(GRMZM2G152135) for maize carotenoids

For *crtRB1*(GRMZM2G152135), there are 22 genes shown expression in the region upstream and downstream of *crtRB1* within 1 MB in 15DAP kernels, with the above 6 kinds of methods to detect the candidate genes for 4 relative traits, that is, *AC* (α-carotene), *BC* (β-carotene), *LUT* (lutein) and *ZEA* (zeaxanthin). The results are shown in Fig. 1, in which abscissa represents 4 traits. The ordinate refers to the effectiveness of each method, which is calculated using Eq. (11). In order to fully explain the experiment result, we illustrate the ranking information of *crtRB1*(GRMZM2G152135) about different phenotypes of 6 kinds of methods, as shown in Table 2. In the table, the number refers to the ranking of the target gene predicted by all the methods. It can be seen that the smaller of the number (the higher of the ranking), the better performance of the method.

**Table 2:** The ranking of *crtRB1*(GRMZM2G152135) about different phenotypes

Methods	RF	SVR_LKF	MI	Elastic-Net	Non-Knockoffs	Knockoffs-NN
Traits						
*AC*	1	1	4	1	1	1
*BC*	1	2	14	1	1	1
*ZEA*	1	2	7	2	1	1
*LUT*	5	12	5	20	13	4

In Fig. 1 and Table 2, we can see the *Knockoffs-NN* method can detect strong signals that *crtRB1*(GRMZM2G152135) having effect of *AC*, *BC*, *ZEA* and *LUT*. *Knockoffs-NN* has the best learning effect than the other 5 kinds of methods. The learning effect of *MI* is the worst of all, and the other 4 methods are in the middle. In addition, we can see the 6 kinds of methods having not very good detection effect for the phenotype of *LUT*. This is related to the dataset correctness of phenotype *LUT*. The methods of *RF*, *SVR_LKF*, *Elastic-Net* and *Non-Knockoffs* can also detect GRMZM2G152135 is affecting *AC*, *BC* and *ZEA* basically. In all, our *Knockoffs-NN* has the best detection accuracy for all the phenotypes. The reason is the importance of each feature gene is evaluated by the weight after training data in *RF*, *SVR_LKF*, *MI*, *Elastic-Net*. These methods only focus on the effect of different genes on the phenotype traits, rather than whether the genes themselves are important or not. But in *Knockoffs-NN* method, the original feature genes and knockoff feature genes are taken as the input of neural network to do training. Each gene can compete with each other in the training process. Through comparing the weight coefficient of original feature genes and knockoff feature genes, *Knockoffs-NN* method determines the importance of each gene. This can increase the stability and reliability of *Knockoffs-NN* method. In addition, the neural network method is suitable for dealing with complex and non-linear data.

### Validation of *lcyE* for maize carotenoids

**Fig. 2:**
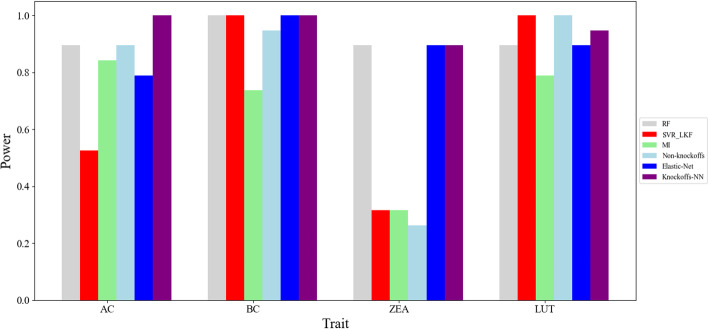
Result of gene *lcyE*(GRMZM2G012966) for maize carotenoids

For *lcyE* (GRMZM2G012966), there are 19 genes shown expression in the region upstream and downstream of *lycE* within 1 MB in 15DAP kernels, with the above 6 kinds of methods to detect the candidate genes for 4 relative traits, that is, *AC* (α-carotene), *BC* (β-carotene), *LUT* (lutein) and *ZEA* (zeaxanthin). The result is shown in Fig. 2. The ranking information of *lcyE*(GRMZM2G012966) about different phenotypes using 6 kinds of methods is illustrated in Table 3.

**Table 3:** The ranking of *lcyE*(GRMZM2G012966) about different phenotypes

Methods	RF	SVR_LKF	MI	Elastic-Net	Non-Knockoffs	Knockoffs-NN
Traits						
*AC*	3	10	4	5	3	1
*BC*	1	1	6	1	2	1
*ZEA*	3	14	1	3	15	3
*LUT*	3	1	5	3	1	2

Through Fig. 2 and Table 3, we can see the *Knockoffs*-*NN* method has the best learning effect on the phenotypes of *AC* and *BC*. For *ZEA*, *MI* has the best detection effect and the learning effect of *Knockoffs*-*NN* is slightly worse than *MI*. But for the phenotype of *LUT*, *MI* has the worst detection effect than the other 5 kinds of methods apparently. In the whole, *Knockoffs*-*NN* has better learning effect than the other 5 kinds of methods for all the phenotypes.

### Validation of *VET4* for maize tocopherol

**Fig. 3:**
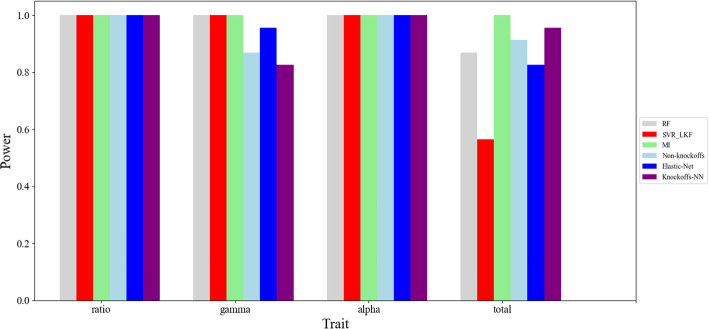
Result of gene *VTE4*(GRMZM2G035213) for maize tocopherol

For *VTE4*(GRMZM2G035213), there are 23 genes shown expression in the region upstream and downstream of *VTE4* within 1 MB in 15DAP kernels, with the above 6 kinds of methods to detect the candidate genes for 4 relative traits, that is *gamma*, *alpha*, *total* and *ratio*. The results are shown in Fig. 3. The ranking information of *VTE4*(GRMZM2G035213) about different phenotypes using 6 kinds of methods is illustrated in Table 4.

**Table 4:** The ranking of *VTE4*(GRMZM2G035213) about different phenotypes

Methods	RF	SVR_LKF	MI	Elastic-Net	Non-Knockoffs	Knockoffs-NN
Traits						
*ratio*	1	1	1	1	1	1
*gamma*	1	1	1	2	4	5
*alpha*	1	1	1	1	1	1
*total*	4	11	1	5	3	2

Through Fig. 3 and Table 4, we can see the 6 kinds of methods have better detection effect for all the phenotypes. The detection effect of *Non-Knockoffs*, *Elastic*-*Net* and *Knockoffs-NN* is slightly worse that *SVR*_*LKF*, *MI and RF* for the phenotype of *gamma*. *MI* has the best learning effect for all the phenotypes. For the phenotype of *total*, the detection accuracy of *MI* is slightly better than *Knockoffs-NN*. This may be related to the linear characteristics of the data. Neural network is suitable for dealing with complex non-linear data, and has better fitting ability. In addition, the knockoffs framework is suitable for the data with independently identically distribution. In all, our *Knockoffs-NN* method can detect strong signals that *VTE4*(GRMZM2G035213) is affecting the 4 phenotypes of maize tocopherol.

### Validation of *ZMGOL* for maize raffinose

**Fig. 4:**
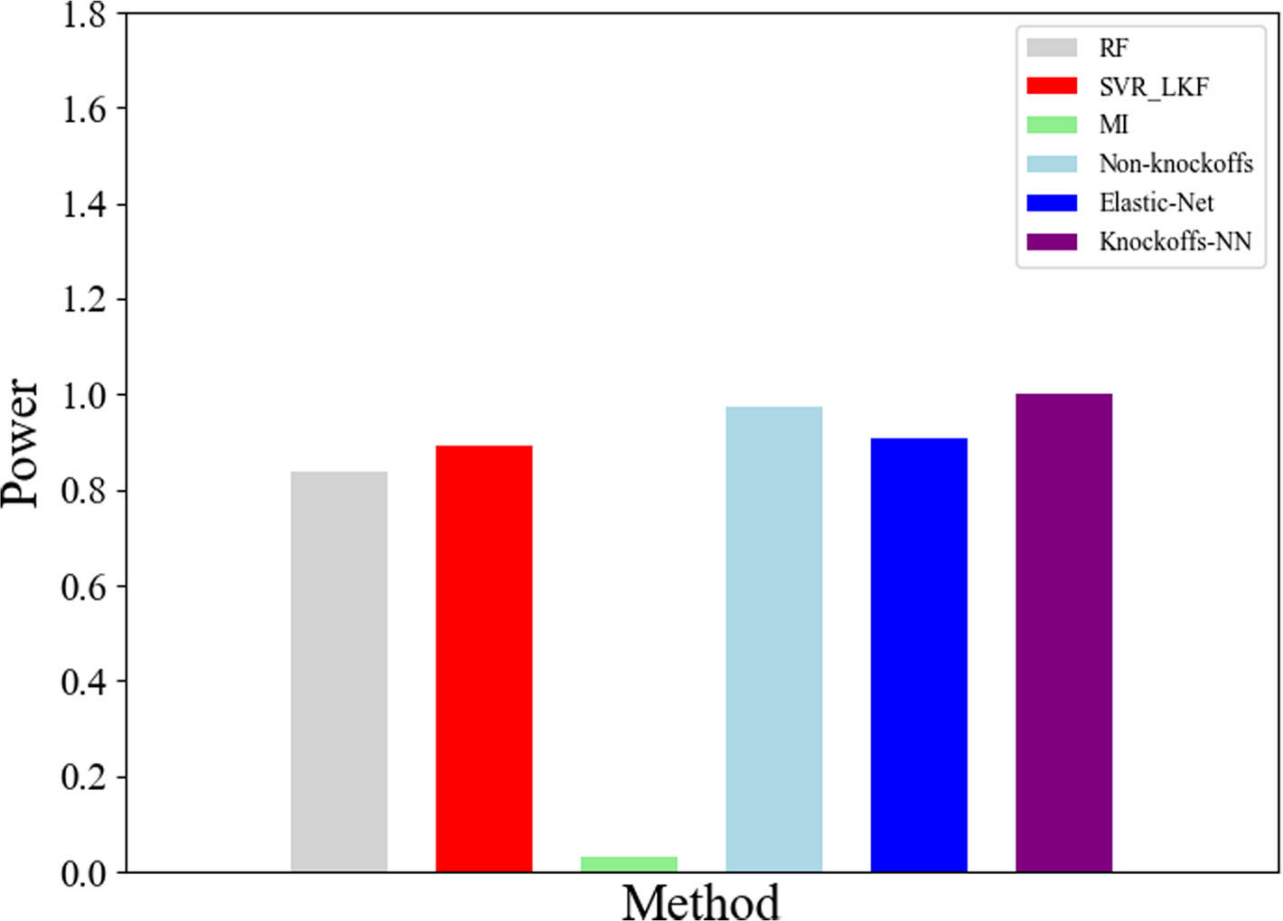
Result of gene *ZmGOL*(GRMZM5G872256) for maize raffinose

For the gene of *ZmGOL* (GRMZM5G872256), there are 215 genes shown expression in the region upstream and downstream of *ZmGOL* within 1 MB, with the above 6 kinds methods to detect the candidate genes for the trait of raffinose family oligosaccharides. The results are shown in Fig. 4. Table 5 shows the ranking information of *ZmGOL* (GRMZM5G872256) about 6 kinds of methods for maize raffinose. The detailed results of *ZmGOL*(GRMZM5G872256) for maize raffinose family oligosaccharides is shown in Table 6. It shows the top-5 genes and the corresponding eigenvalues that have been calculated using different methods.

**Table 5:** The ranking of *ZmGOL*(GRMZM5G872256) about maize raffinose

Methods	RF	SVR_LKF	MI	Elastic-Net	Non-Knockoffs	Knockoffs-NN
Trait						
M71	36	24	209	21	7	1

**Table 6:** The results of *ZmGOL*(GRMZM5G872256) about top-5 genes

Methods	RF	SVR_LKF	MI	Non- Knockoffs	Elsatic- Net	Knockoffs-NN
Number						
1	GRMZM2G092174 (0.048)	GRMZM2G005984 (0.396)	GRMZM2G043295 (0.126)	GRMZM2G121360 (0.529)	GRMZM2G121360 (0.420)	GRMZM5G872256 (0.00358520178299)
2	GRMZM2G060842 (0.033)	GRMZM2G022686 (0.221)	GRMZM2G092174 (0.108)	GRMZM5G875954 (0.411)	GRMZM2G102382 (0.377	GRMZM5G877547 (0.00290219524756)
3	GRMZM2G129815 (0.027)	GRMZM2G121360 (0.205)	GRMZM2G129815 (0.090)	GRMZM2G134107 (0.390)	GRMZM2G005984 (0.376)	GRMZM2G134471 (0.0028602059655)
4	GRMZM2G022398 (0.022)	GRMZM2G040268 (0.164)	GRMZM2G121360 (0.088)	GRMZM5G850567 (0.323)	GRMZM2G134107 (0.359)	GRMZM2G700004 (0.00263825481301)
5	GRMZM5G875954 (0.022)	GRMZM2G181551 (0.161)	GRMZM2G317262 (0.087)	GRMZM5G872256 (0.304)	GRMZM2G415117 (0.320)	GRMZM5G875954 (0.00259085517355)

From Tables 5, 6 and Fig. 4, we can see the learning effect of various methods are quite different on the target gene *GRMZM5G872256*. It ranked 36th in *RF* method, 24th in *SVR_LKF* method, 209th in *MI* method, 5th in *Non-Knockoffs*, 21th in *Elastic-Net* method and 1st in our *Knockoffs-NN* method. It can be seen that the neural network related methods (*Knockoffs-NN* and *Non-Knockoffs*) have better detection effect. This is related to the fact that the neural network method is suitable for dealing with complex, non-linear data with a large number of features. In addition, this dataset has the characteristics of non-linear and with independently identically distribution, which is suitable for the *Knockoffs-NN* method. Therefore, our *Knockoffs-NN* method has the best detection effect obviously.

### Validation of human breast dataset

**Fig. 5:**
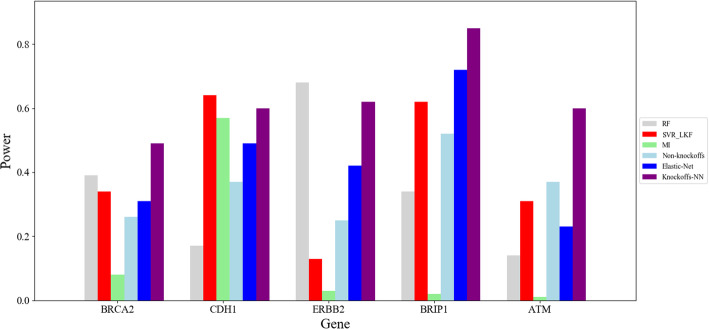
Results of human breast cancer dataset

There are 286 samples and 13,698 genes in the human breast cancer data (https://www.ncbi.nlm.nih.gov/geo/query/acc.cgi?acc=GSE2034). On the basis of the top-10 genes available at https://www.genecards.org/ of breast cancer, we selected 90 genes from 13,698 genes to do experiment. We take the average value of 10 experiment results to do comparison. Figure 5 shows the result of top-5 genes about human breast cancer dataset. The gene ranking information about the top-5 genes using 6 kinds of methods is illustrated in Table 7.

**Table 7:** The gene ranking of human breast cancer dataset

Methods	RF	SVR_LKF	MI	Elastic-Net	Non-Knockoffs	Knockoffs-NN
Ranking						
RCA2	62	67	62	70	75	52
*DH1*	84	37	84	52	64	41
*RBB2*	33	88	33	59	76	39
*BRIP1*	67	39	67	29	49	16
*ATM*	87	70	87	78	64	41

Because the number of samples and feature genes is 107 and 13,698 respectively in this dataset, we use random sampling method to include the other 90 unrelated genes to do the experiment. However, due to the small number of samples and the large randomness of the selected genes, the learning effect of 6 kinds of methods is not very good. But we still can see *Knockoffs-NN* performs better than other methods in the whole.

## Discussion

According to the above experiment results, it can be seen that our *Knockoffs-NN* method is more stable than other 5 kinds of methods. And it has the best detection effect in all the 6 kinds of methods. Although other methods may be performed well on one trait, but they are less stable on the whole. The reason is that these methods only focus on the effect of different genes on the phenotype traits, rather than whether the genes themselves are important or not. While our *Knockoffs-NN* method constructs the knockoff feature genes of the original feature genes in the neural network. Then it takes the original feature genes and knockoff feature genes as the input of neural network to do training. In the training process, each gene can not only compete with each other but also compete with its knockoff feature gene. This method can determine the importance of each gene through comparing the weight of original feature genes and knockoff feature genes. That is to say, this method can evaluate the importance of genes for the phenotype traits with the help of the knockoff feature genes. Therefore, the *Knockoffs-NN* method can evaluate whether the genes themselves are important or not. In addition, the neural network method is suitable for dealing with complex and non-linear data.

## Conclusions

Genes are genetic information that controls biological traits. Mining genes affecting specific phenotypic trait has important research significance for crop quality improvement, diagnosis and treatment of human diseases. At present, more and more studies have used machine learning related methods for gene selection. These methods can not only mine candidate genes associated with phenotypic traits efficiently, but also greatly reduce the time and cost of biology research. This work proposes a kind of embedded gene selection method based on knockoffs optimizing neural network. This method introduces the idea of knockoffs into the neural network construction. It constructs the knockoff feature genes of the original feature genes, and realizes the feature gene selection by calculating the weight coefficient of each feature gene after training. This method not only make each feature gene to compete with each other, but also make each feature gene compete with its knockoff feature gene. We mainly describe the specific process of constructing knockoff feature genes. This method can deal with the complex relationships between genes and phenotypes, and then mine candidate genes affecting specific phenotypic traits. The effectiveness of the method is validated using the real datasets, including maize carotenoids, tocopherol methyltransferase, raffinose family oligosaccharides and human breast cancer. The experiment results show that our knockoffs optimizing neural network method has better gene selection effect than the other 5 kinds of existing methods. Specially, the proposed method is suitable to process the complex non-linear data with independently identically distribution. This characteristic is just for dealing with the data of gene expression and phenotypes.

Although the knockoffs optimizing neural network has a good learning effect on each dataset, there are still some shortcomings need to do further research. Firstly, the neural network is prone to overfitting during the training process due to the high-dimensional characteristic of the gene expression data. This may lead to the selected feature genes not accurate enough. We plan to select a part of candidate genes firstly from the original genes using the traditional gene selection method, and then further to select the target genes by our proposed *Knockoffs-NN* method. Secondly, we found that when the sample number is 10 times more than gene feature number, our method has better normalization ability. But in the biological area, the number of genes may be very large and our method may not perform well enough. In addition, the source code that is currently used to generate knockoff genes can only handle nonsingular matrices with the number of samples is larger than the number of features. We will further to do improvement according to the framework of knockoffs optimizing neural network. We endeavor to process the dataset with number of samples is less than the number of gene features, and then conduct experiments on new datasets. Finally, how to handle the multi-classification tasks, for example, gene selection for multiple phenotypes, is still a problem to be solved.

## Methods

### Neural network

Neural network has better fitting ability when to deal with the complex non-linear data by utilizing multiple hidden layers and activation functions. The activation function can increase the fitting ability to the non-linear data. The most commonly used activation function is the Rectified Linear Units (*ReLU*) function, as shown in Eq. (2).

2 $$ f(x)=\max \left(0,x\right) $$

Different loss functions are used for different learning problems. For example, the mean square error loss function is generally used to deal with regression problems, as shown in Eq. (3). In the equation, *y* denotes the true value, *f*(*x*, *W*) denotes the predicted value after training. *W* represents parameters in the model of neural network. Firstly, we initialize the parameter *W* randomly and then update it through the gradient descent method.

3 $$ cost={\left(y-f\left(x,W\right)\right)}^2 $$

We use softmax function to deal with multi-classification problems, as shown in Eq. (4). In the equation, *C* represents the number of categories and *W* represents the weight matrix.

4 $$ cost=-\sum \limits_k{Y}_k\log \left( softmax\left({W}^{\mathrm{T}}{X}_k\right)\right), softmax\left({w}_c^{\mathrm{T}}x\right)=\frac{\exp \left({w}_c^{\mathrm{T}}x\right)}{\sum \limits_{c=1}^C\exp \left({w}_c^{\mathrm{T}}x\right)}, $$

### Knockoff feature

For each sample in the dataset, we use *x* = (*X*_*1*_,…,*X*_*k*_) to represent the original feature (gene) and $\tilde{x}={\left({\tilde{X}}_1,\cdots, {\tilde{X}}_k\right)}^T$ to represent the knockoff features. The original feature *x* and knockoff feature $\tilde{x}$ need to satisfy the following two conditions [24].

(1). For any subset *S* ⊂ {1, ⋯, *k*}, it needs to satisfy ${\left(x,\tilde{x}\right)}_{swap(S)}\overset{d}{=}\left(x,\tilde{x}\right)$, where *swap*(*S*) represents exchanging *j* and  for any *j* ⊂ *S*.  represents the same data distribution. For example, *n* = 2, *s* = {2},we can get ${\left({X}_1,{X}_2,{\tilde{X}}_1,{\tilde{X}}_2\right)}_{swap\left(\left\{2\right\}\right)}\overset{d}{=}\left({X}_1,{\tilde{X}}_2,{\tilde{X}}_1,{X}_2\right)$.

(2). $\tilde{x}$ ╨ *Y* ∣ *x*. It represents $\tilde{x}$ independent of *Y* in the condition of given *x*.

### Knockoffs-NN approach

#### The framework of knockoffs-NN

**Fig. 6:**
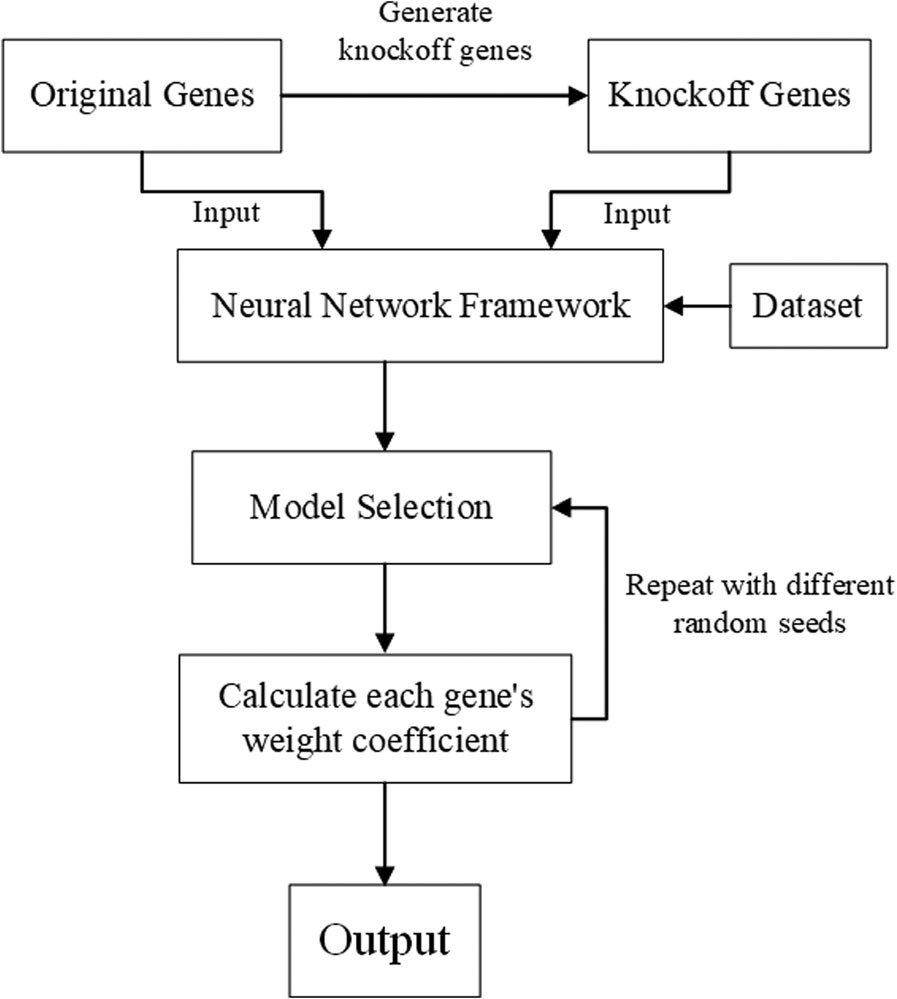
The framework of our method

The framework of our method is shown in Fig. 6. In Fig. 6, the knockoff gene features are generated based on the original genes firstly. Taken the original genes and knockoff genes as the input and phenotype trait as output, then we use the neural network to realize the model training. We select the optimal neural network parameters and realize model selection based on the validation dataset. Finally, we calculate the weight coefficient of each gene after the training is completed. In order to insure the accuracy, we repeat the above process several times with different random seeds and obtain the average weight coefficient of each gene.

### Constructing knockoff features

#### Constructing accurate knockoff features

We can construct knockoff features according to the two conditions described in the section of Knockoff feature. Supposing *x* ∼ Ν(0, Σ), Σ represents covariance matrix and Σ ∈ ℝ^*p* × *p*^. The joint distribution of *x* and $\tilde{x}$ satisfying the above two conditions is shown in Eq. (5) [24].

5 $$ \left(x,\tilde{x}\right)\sim N\left(0,G\right),G=\left(\begin{array}{cc}\Sigma & \Sigma -\mathit{\operatorname{diag}}\left\{p\right\}\\ {}\Sigma -\mathit{\operatorname{diag}}\left\{p\right\}& \Sigma \end{array}\right) $$

In Eq. (5), *diag*{*p*} is an arbitrary diagonal matrix selected in a way that the joint covariance matrix *G* is positive semidefinite. We can obtain the knockoff feature $\tilde{x}$ from conditional distribution sampling of $\left.\tilde{x}\right|x$, as shown in Eq. (6) [24].

6 $$ \left.\tilde{x}\right|x\sim \mathrm{N}\left(x-\mathit{\operatorname{diag}}\left\{p\right\}{\Sigma}^{-1}x,2\mathit{\operatorname{diag}}\left\{p\right\}-\mathit{\operatorname{diag}}\left\{\mathrm{p}\right\}{\Sigma}^{-1}\mathit{\operatorname{diag}}\left\{p\right\}\right) $$

More generally, if the data does not obey the Gaussian distribution, the knockoff features can be constructed using the following methods. Any knockoff features $\left({\tilde{X}}_1,\cdots, {\tilde{X}}_k\right)$ of (*X*_1_, ⋯, *X*_*k*_) satisfy the two conditions. If the elements in vector *X* are independent, then any independent copy of *X* needs to satisfy the two conditions. That is to say, any $\tilde{X}$ that is independently sampled from the same joint distribution as *X* satisfies these two conditions. We can construct the knockoff feature $\tilde{\mathrm{X}}$ using Algorithm 1 [24].

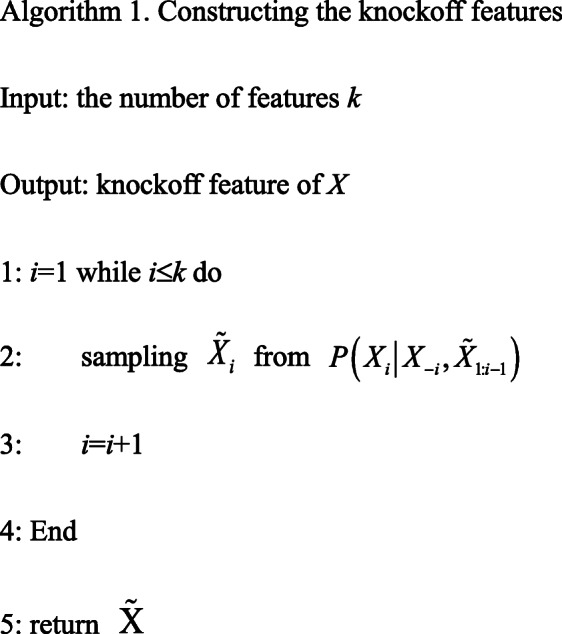



In Algorithm 1, $P\left(\left.{X}_i\right|{X}_{-i},{\tilde{X}}_{1:i-1}\right)$ represents the conditional distribution of *X*_*i*_ given $\left({X}_{-i},{\tilde{X}}_{1:i-1}\right)$. *X*_−*i*_ represents all the features except for the *i*-th feature. Firstly, we sample ${\tilde{X}}_1$ from the conditional distribution of *P*(*X*_1_|*X*_2_). Then we can get $P\left({X}_{1:2},{\tilde{X}}_1\right)$ and $P\left(\left.{X}_2\right|{X}_1,{\tilde{X}}_1\right)$ can be calculated. In the next iteration, we sample ${\tilde{X}}_2$ from the conditional distribution of $P\left(\left.{X}_2\right|{X}_1,{\tilde{X}}_1\right)$. Then we can get the knockoff features ${\tilde{X}}_1,{\tilde{X}}_2$ according to Algorithm 1.

#### Constructing approximate knockoff features

According to Algorithm 1, we can obtain the accurate knockoff features. But it will consume a lot of time because the conditional distribution needs to be calculated at each step. To simplify this process, we can use the approximate knockoff feature construction method [37].

Constructing approximate knockoff features no longer requires ${\left(X,\tilde{X}\right)}_{swap(S)}$ and $\left(X,\tilde{X}\right)$ having the same distribution, but it requires them to have the same mean and covariance. It is easy to ensure the mean having the same value. Eq. (7) needs to be satisfied to ensure the covariance having the same value.

7 $$ \operatorname{cov}\left(X,\overline{X}\right)=G,G=\left(\begin{array}{cc}\sum & \sum -\mathit{\operatorname{diag}}\left\{p\right\}\\ {}\sum -\mathit{\operatorname{diag}}\left\{p\right\}& \sum \end{array}\right) $$

It is necessary to select parameter *s* to produce a semi-positive definite covariance matrix. We can construct approximate knockoff features using the following two steps.

Step 1: Select the approximate value ∑_*approx*_ of ∑ and solve the optimization problem shown in Eq. (8).

8 $$ \operatorname{minimize}\;{\sum}_j\left|1-{\hat{p}}_j\right|\kern0.37em \;\mathrm{subject}\ \mathrm{to}\kern0.24em {\hat{p}}_j\ge 0,\mathit{\operatorname{diag}}\left\{\hat{p}\right\}\le 2{\sum}_{approx} $$

Step 2: Solve the optimization problem shown in Eq. (9).

9 $$ \operatorname{maximize}\kern0.24em \upgamma \kern0.24em \mathrm{subject}\ \mathrm{to}\kern0.24em \mathit{\operatorname{diag}}\left\{\upgamma \hat{p}\right\}\le 2\Sigma $$

The returning value $\upgamma \hat{p}$ is the selected parameter *p* in Eq. (6). Among them, step 2 can be solved quickly using the binary search method (*γ* ∈ [0, 1]). The conditional distribution $\left.\tilde{x}\right|x$ can be obtained using Eq. (6), and thus to get the knockoff feature genes.

In general, we can choose ∑_*approx*_ as the *m*-block diagonal approximation of ∑. Then we can divide the operation of step 1 into *m* sub-problems, which are smaller and easier to calculate and can be processed in parallel. If the approximation is accurate enough, larger values of γ can be got. It means that the approximation value and the exact value of the knockoff variables are identical.

#### Knockoff filter construction

After obtaining the knockoff feature gene $\tilde{x}$, we can select important feature genes by sorting the knockoff statistic variables of ${W}_{\mathrm{j}}={f}_j\left({Z}_j,{\tilde{Z}}_j\right)$ [23]. *f*_*j*_(.,.) represents the anti-symmetric function with ${f}_j\left({Z}_j,{\tilde{Z}}_j\right)=-{f}_j\left({\tilde{Z}}_j,{Z}_j\right)$. It should be noted that the measurement of feature importance and the construction of knockoff statistic *W* are not same for different fitting model algorithms. Strictly speaking, the knockoff variables should satisfy the flipping attribute. It means that any exchange of *X*_*j*_ and ${\tilde{X}}_j$ will only change the sign of *W*_*j*_, but it will not change the sign of other variables of *W*_*k*_ (*k* ≠ *j*). It is conceivable that if a feature gene *j* is important, its corresponding knockoff variable *W*_*j*_ will be a large positive value. On the contrary, if a feature gene *j* is not important, the value of *W*_*j*_ is close to zero.

On the basis of the knockoff variable *W*_*j*_, we rank it according to |*W*_*j*_|. Next, we select feature genes on the basis of the threshold *T*, which can be calculated using two methods shown in Eq. (10).

10 $$ T=\min \left\{t\in \omega, \frac{\left|\left\{j:{W}_j\le -t\right\}\right|}{\left|\left\{j:{W}_j\ge t\right\}\right|}\le q\right\},{T}_{+}=\min \left\{t\in \omega, \frac{1+\left|\left\{j:{W}_j\le -t\right\}\right|}{1\vee \left|\left\{j:{W}_j\ge t\right\}\right|}\le q\right\} $$

In Eq. (10), |{}| represents the set size. *ω* = {|*W*_*j*_| : 1 ≤ *j* ≤ *p*}\{0}, and it represents the unique set of non-zero values based on |*W*_*j*_|. |*W*_*j*_| represents the absolute value of *W*_*j*_. *q* represents the false discovery rate (*FDR*) we expected, and 1 ∨ |{*j* : *W*_*j*_ ≥ *t*}| represents max(1, |{*j* : *W*_*j*_ ≥ *t*}|).

In all, we can get the knockoff feature set *W* after using the neural network to fit the model. Then we select all the feature genes *j* that satisfy *W*_*j*_ ≥ *T* as the final selected important genes, which satisfying the condition that *FDR* is less than or equal to *q*.

#### Knockoffs optimizing neural network

Neural networks generally contain one input layer, multiple hidden layers and one output layer. In neural network, the layers are generally fully connected. In order to make the training results interpretable and discover the key genes in neural networks, we use knockoffs into neural network construction and select important genes affecting specific phenotypic traits according to the weight of each gene after training. Using the idea of *DeepInk* proposed in [23], we introduce a coupling layer which contains *p* filters. Each filter *j* connects the original features (genes) *X*_*j*_ and knockoff features (genes) ${\tilde{X}}_j$ by weights *z*_*j*_ and ${\tilde{z}}_j$. *z*_*j*_ and ${\tilde{z}}_j$ have the same initial values. In the process of training, *z*_*j*_ and ${\tilde{z}}_j$ will compete with each other. After training, if the feature gene *j* is important, the value of *z*_*j*_ will be much larger than ${\tilde{z}}_j$. On the contrary, if feature gene *j* is not important, then the value of *z*_*j*_ will be close to ${\tilde{z}}_j$. Except for each feature gene competing with its knockoff feature gene, it is also necessary to allow each feature gene to compete with each other. In order to achieve this goal, we use linear activation function in the coupling layer. After passing through the coupling layer, we connect the output of *p* values to a multi-layer perceptron (*MLP*) to learn the function from input feature genes to output *Y*. In the multilayer perceptron, we have multiple activation layers with alternating linear and non-linear changes. Each layer learns the mapping from input to hidden layer, and the last layer can learn the mapping of output *Y* (phenotype).

**Fig. 7:**
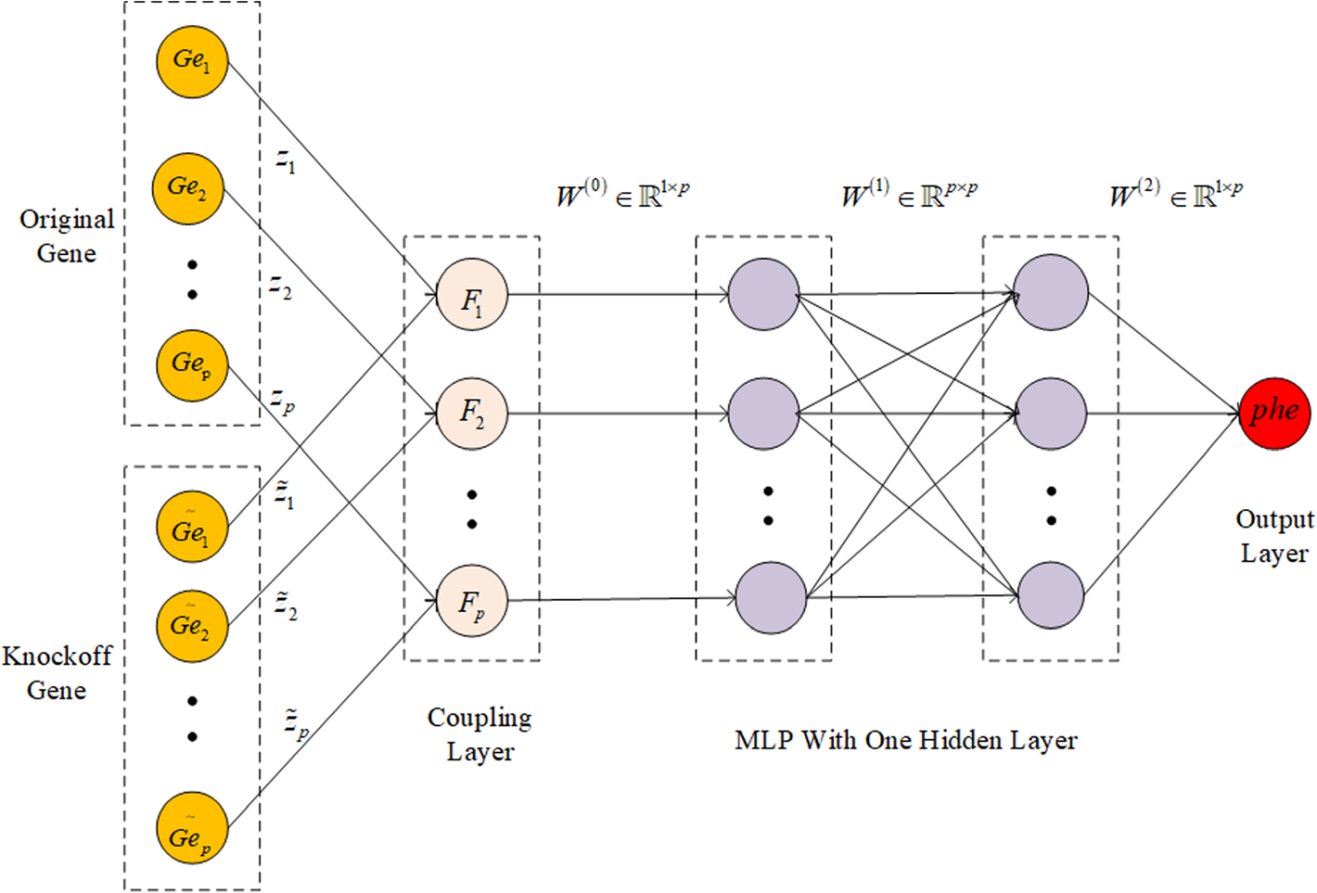
The gene selection framework of knockoff optimizing neural network

When there is 1 hidden layer and each layer contains *p* neurons, the network structure is shown in Fig. 7. We choose *ReLU* activation function, *L1* regularization and mean square error loss function. In Fig. 7, *Ge*_*1*_, *Ge*_*2*_,…,*Ge*_*p*_ represent the input of the original genes. $\overset{\sim }{Ge_1},\overset{\sim }{Ge_2},\dots, \overset{\sim }{Ge_p}$ represent the constructed knockoff feature genes, and *Phe* represents phenotypic trait. *z*_*j*_ and ${\tilde{z}}_j$ represent the weight vectors of the input layer connecting the coupling layer, and their initial values are same to ensure fair competition. *W*^(0)^ ∈ ℝ^p × 1^ represent the weight vector of coupling layer connecting multilayer perceptron. *W*^(1)^ ∈ ℝ^p × *p*^ and *W*^(2)^ ∈ ℝ^p × 1^ represent the weight matrix of the input layer connecting the hidden layer and the hidden layer connecting the output layer respectively.

As shown in Fig. 2, the importance measurement of *Z*_*j*_ and ${\tilde{Z}}_j$ are determined by the following two criteria. (1) The relative importance of *Ge*_*j*_ and its knockoff feature $\overset{\sim }{Ge_j}$, represented by filter weights of ***z*** = (*z*_1_, ⋯, *z*_*j*_)^*T*^ and $\tilde{\boldsymbol{z}}={\left({\tilde{z}}_1,\cdots, {\tilde{z}}_j\right)}^T$. (2) The relative importance of the *j*th feature gene in *p* features, represented by a weight matrix, ***w =*** W^(0)^ ⊙ (*W*^(1)^*W*^(2)^*W*^(3)^). ⊙ represents Hadamard product. Then we define *Z*_*j*_ and ${\tilde{Z}}_j$ using Eq. (11).

11 $$ {Z}_j={z}_j\times {\boldsymbol{w}}_{\boldsymbol{j}},{\tilde{Z}}_j={\tilde{z}}_j\times {\boldsymbol{w}}_{\boldsymbol{j}} $$

Then we can get the knockoff variables using ${W}_j={Z^2}_j-{{\tilde{Z}}^2}_j$. The gene selection is performed using the method described in the section of constructing Knockoff features. It should be noted that we need to find the best network structure and hyperparameters for fitting about different data. After determining the network structure and hyperparameters, we can obtain the knockoff feature genes through training the model. Then it performs the final feature gene selection according to the threshold *T*.

## Supplementary information

**Additional file 1.** Supplementary manuscript.docx. The file includes seven figures (Figure 1 - Figure 7) and seven tables (Table 1 - Table 7).

**Additional file 2.** Revision Response.

**Additional file 3.** Dataset.rar (Dataset and results).

**Additional file 4.** Knockoffs-NN.rar (Source code package).

## Data Availability

All data generated or analyzed during this study are included in this published article. All the data for this work is available at http://122.205.95.139/Knockoffs-NN/Dataset.rar. All the source code for this work is available at http://122.205.95.139/Knockoffs-NN/Knockoffs-NN.rar.

## Abbreviations

GWAS: Genome-Wide Association Study
QTL: Quantitative Trait Locus
LASSO: Least absolute shrinkage and selection operator
ReLU: Rectified linear units
CFS: Correlation-based feature selection
SVM: Support vector machine
MLP: Multi-layer perceptron
AMP: Association mapping panel
CFS: Correlation-based feature selection
ABC: Artificial bee colony
RF: Random forest
SVR_LKF: SVR linear kernel function
MI: Mutual information
