# Supplementary material for: An embedded gene selection method using knockoffs optimizing neural network
Source: BMC Bioinformatics. 2020 Sep 22;21:414. doi: 10.1186/s12859-020-03717-w (PMC7510330; doi:10.1186/s12859-020-03717-w)
Supplement: Supplementary file 1 — Additional file 1. Supplementary manuscript.docx. The file includes seven figures (Figure 1 - Figure 7) and seven tables (Table 1 - Table 7). [file 12859_2020_3717_MOESM1_ESM.docx]

**An Embedded Gene Selection Method Using Knockoffs Optimizing Neural Network**

Juncheng Guo^1,2,3^, Min Jin^4^, Yuanyuan Chen^4^, Jianxiao Liu^1,4,*^

^1^Hubei Key Laboratory of Agricultural Bioinformatics, College of Informatics, Huazhong Agricultural University, Wuhan, 430070

^2^Institute of Information Engineering, Chinese Academy of Sciences, Beijing 100093, China

^3^School of Cyber Security, University of Chinese Academy of Sciences, Beijing 100093, China

^4^National Key Laboratory of Crop Genetic Improvement, Huazhong Agricultural University, Wuhan, 430070

E-mail addresses:

Juncheng Guo: yuexiaozhuoke@gmail.com (J. Guo)

Min Jin: 834245464@qq.com (M. Jin)

Yuanyuan Chen: yychentamu@163.com (Y. Chen)

Jianxiao Liu: liujianxiao321@163.com (J. Liu)

^*^Correspondence: Jianxiao Liu ([liujianxiao321@163.com](mailto:liujianxiao321@163.com))

The file includes seven figures (Figure 1 – Figure 7) and seven tables (Table 1 –Table 7). All the data is available at http://122.205.95.139/Knockoffs-NN/Dataset.rar. All the source code is available at http://122.205.95.139/Knockoffs-NN/Knockoffs-NN.rar.

## Figures


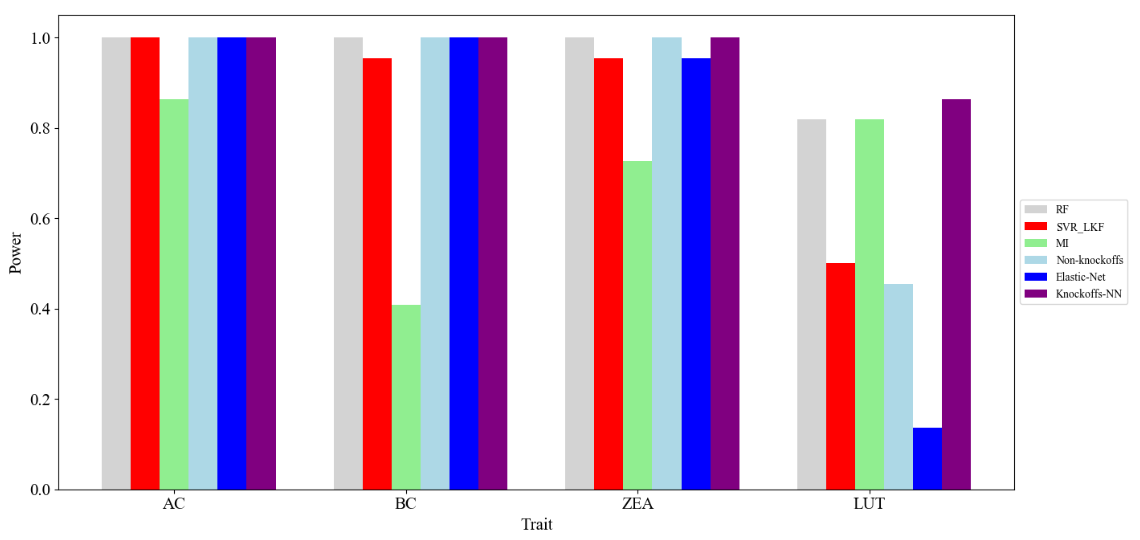


Figure 1. Result of gene *crtRB1*(GRMZM2G152135) for maize carotenoids


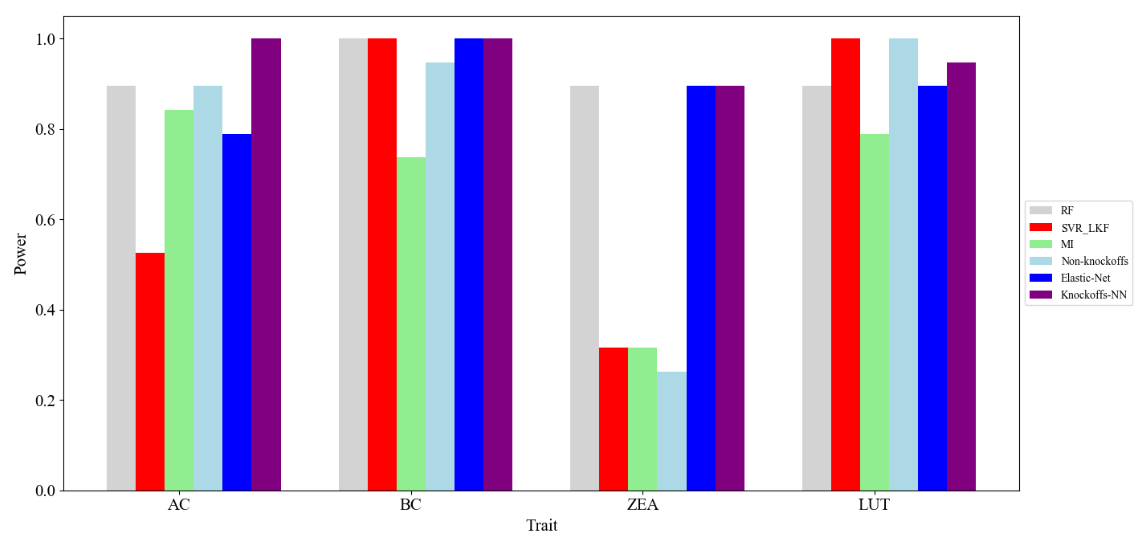


Figure 2. Result of gene *lcyE*(GRMZM2G012966) for maize carotenoids


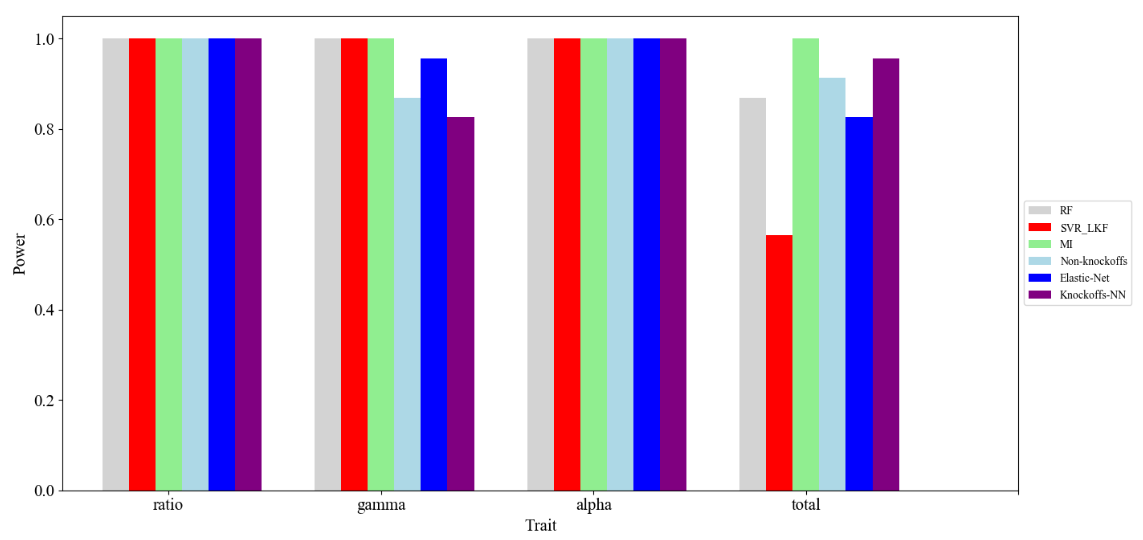


Figure 3. Result of gene *VTE4*(GRMZM2G035213) for maize tocopherol


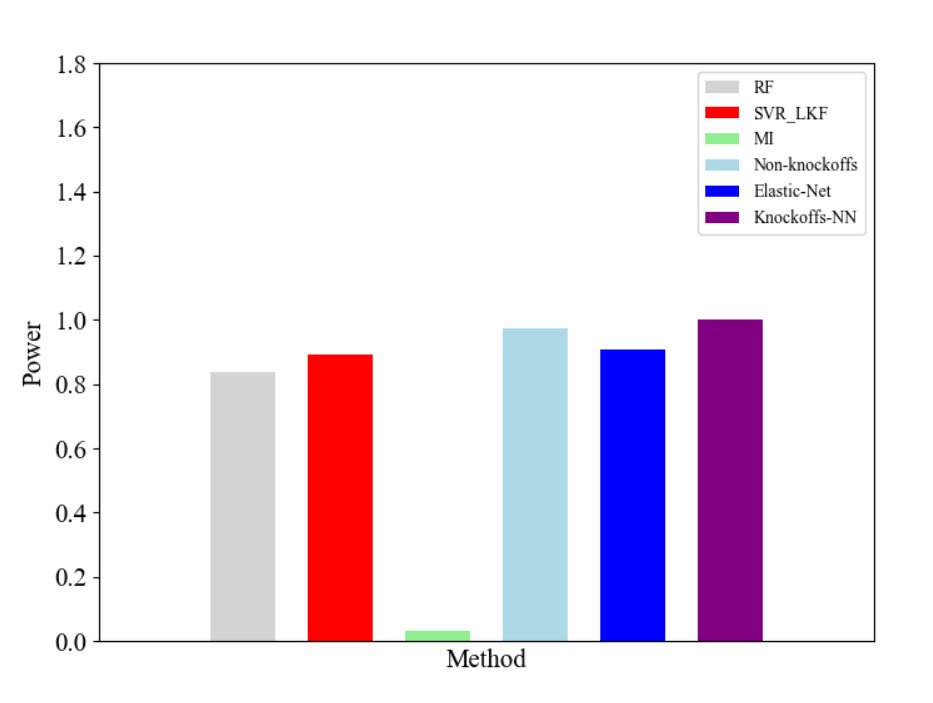


Figure 4. Result of gene *ZmGOL* (GRMZM5G872256) for maize raffinose


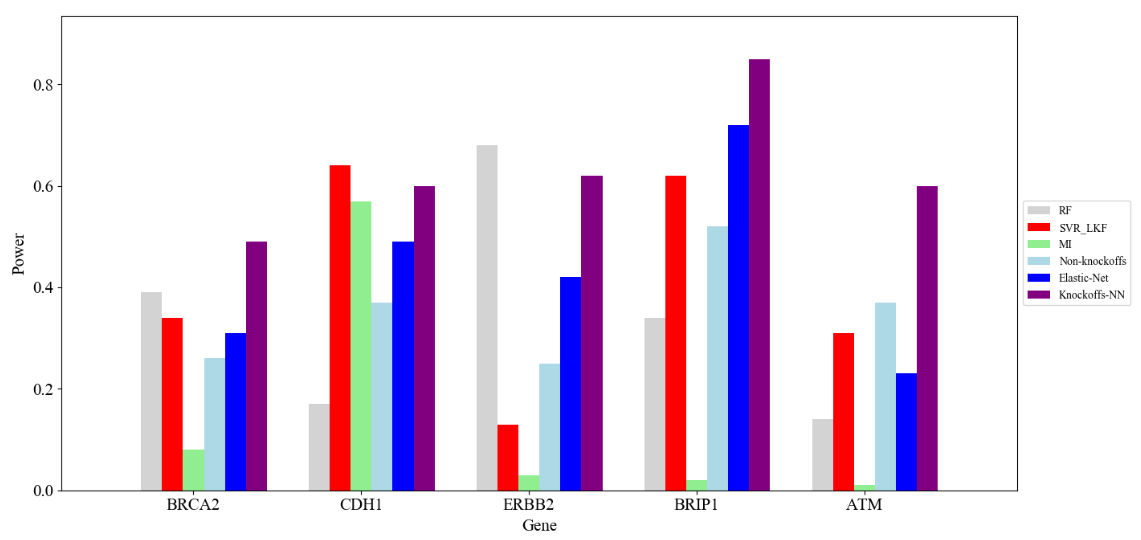


Figure 5. Results of human breast cancer dataset

Figure 6. The framework of our method


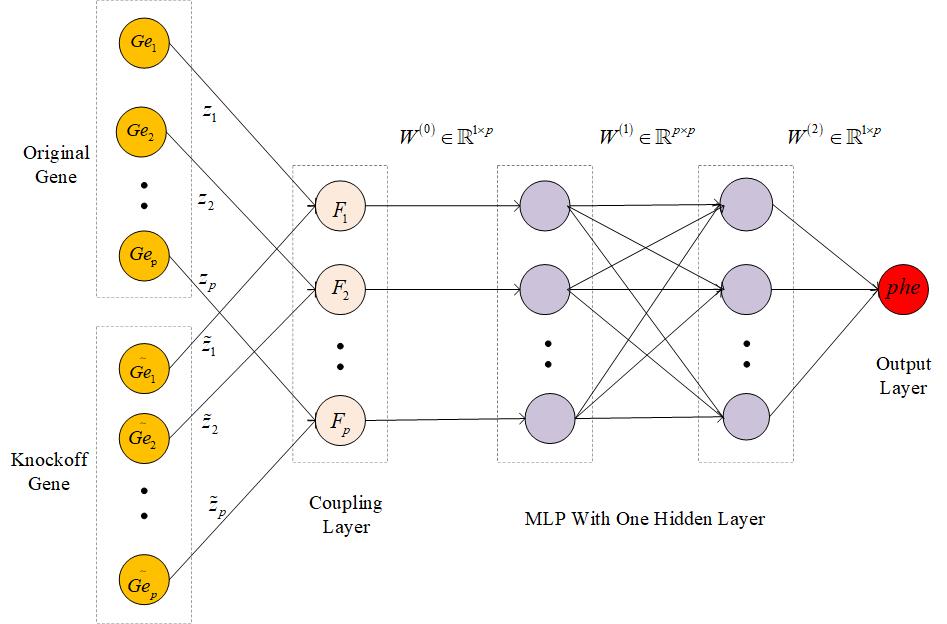


Figure 7. The gene selection framework of knockoff optimizing neural network

## Tables

Table 1. Parameters setting in the experiment

| Parameter setting | Value |
| --- | --- |
| Activation function | ReLU |
| Regularization | L1 |
| Loss function | Mean square error (MSE) |
| Optimization | Batch gradient descent and Adam (recommend mini-batch for large samples) |
| Number of hidden layer | 1 |
| Number of hidden layer neurons (genes) | Number of genes |
| Learning rate | 0.0001 |

Table 2. The ranking of *crtRB1*(GRMZM2G152135) about different phenotypes

| Methods  Traits | RF | SVR_LKF | MI | Elastic-Net | Non-Knockoffs | Knockoffs-NN |
| --- | --- | --- | --- | --- | --- | --- |
| *AC* | 1 | 1 | 4 | 1 | 1 | 1 |
| *BC* | 1 | 2 | 14 | 1 | 1 | 1 |
| *ZEA* | 1 | 2 | 7 | 2 | 1 | 1 |
| *LUT* | 5 | 12 | 5 | 20 | 13 | 4 |

Table 3. The ranking of *lcyE*(GRMZM2G012966) about different phenotypes

| Methods  Traits | RF | SVR_LKF | MI | Elastic-Net | Non-Knockoffs | Knockoffs-NN |
| --- | --- | --- | --- | --- | --- | --- |
| *AC* | 3 | 10 | 4 | 5 | 3 | 1 |
| *BC* | 1 | 1 | 6 | 1 | 2 | 1 |
| *ZEA* | 3 | 14 | 1 | 3 | 15 | 3 |
| *LUT* | 3 | 1 | 5 | 3 | 1 | 2 |

Table 4. The ranking of *VTE4*(GRMZM2G035213) about different phenotypes

| Methods  Traits | RF | SVR_LKF | MI | Elastic-Net | Non-Knockoffs | Knockoffs-NN |
| --- | --- | --- | --- | --- | --- | --- |
| *ratio* | 1 | 1 | 1 | 1 | 1 | 1 |
| *gamma* | 1 | 1 | 1 | 2 | 4 | 5 |
| *alpha* | 1 | 1 | 1 | 1 | 1 | 1 |
| *total* | 4 | 11 | 1 | 5 | 3 | 2 |

Table 5. The ranking of *ZmGOL*(GRMZM5G872256) about maize raffinose

| Methods  Trait | RF | SVR_LKF | MI | Elastic-Net | Non-Knockoffs | Knockoffs-NN |
| --- | --- | --- | --- | --- | --- | --- |
| M71 | 36 | 24 | 209 | 21 | 7 | 1 |

Table 6. The results of *ZmGOL*(GRMZM5G872256) about top-5 genes

| Methods  Number | RF | SVR_LKF | MI | Non-  Knockoffs | Elsatic-  Net | Knockoffs-NN |
| --- | --- | --- | --- | --- | --- | --- |
| 1 | GRMZM2G092174  (0.048) | GRMZM2G005984  (0.396) | GRMZM2G043295  (0.126) | GRMZM2G121360  (0.529) | GRMZM2G121360  (0.420) | GRMZM5G872256  (0.00358520178299) |
| 2 | GRMZM2G060842  (0.033) | GRMZM2G022686  (0.221) | GRMZM2G092174  (0.108) | GRMZM5G875954  (0.411) | GRMZM2G102382  (0.377 | GRMZM5G877547  (0.00290219524756) |
| 3 | GRMZM2G129815  (0.027) | GRMZM2G121360  (0.205) | GRMZM2G129815  (0.090) | GRMZM2G134107  (0.390) | GRMZM2G005984  (0.376) | GRMZM2G134471  (0.0028602059655) |
| 4 | GRMZM2G022398  (0.022) | GRMZM2G040268  (0.164) | GRMZM2G121360  (0.088) | GRMZM5G850567  (0.323) | GRMZM2G134107  (0.359) | GRMZM2G700004  (0.00263825481301) |
| 5 | GRMZM5G875954  (0.022) | GRMZM2G181551  (0.161) | GRMZM2G317262  (0.087) | GRMZM5G872256  (0.304) | GRMZM2G415117  (0.320) | GRMZM5G875954  (0.00259085517355) |

Table 7. The gene ranking of human breast cancer dataset

| Methods  Ranking | RF | SVR_LKF | MI | Elastic-Net | Non-Knockoffs | Knockoffs-NN |
| --- | --- | --- | --- | --- | --- | --- |
| RCA2 | 62 | 67 | 62 | 70 | 75 | 52 |
| *DH1* | 84 | 37 | 84 | 52 | 64 | 41 |
| *RBB2* | 33 | 88 | 33 | 59 | 76 | 39 |
| *BRIP1* | 67 | 39 | 67 | 29 | 49 | 16 |
| *ATM* | 87 | 70 | 87 | 78 | 64 | 41 |
