# Supplementary material for: An embedded gene selection method using knockoffs optimizing neural network
Source: BMC Bioinformatics. 2020 Sep 22;21:414. doi: 10.1186/s12859-020-03717-w (PMC7510330; doi:10.1186/s12859-020-03717-w)
Supplement: Supplementary file 2 — Additional file 2. Revision Response. [file 12859_2020_3717_MOESM2_ESM.docx]

Dear Mr./Ms. editor(s):

We appreciate your valuable comments and tried our best to address them in the revised version of our paper. We made ​​the following modifications.

1. Please remove any files from the file inventory that you do not wish to see published.

**Answer:** Thank you. Yes, we have removed the files that will not be published in final publication.

2. In accordance with BMC editorial policy we require all research involving plants to have voucher specimens deposited to ensure repeatability of the study (https://www.biomedcentral.com/getpublished/editorial-policies#research+involving+plants). Please clarify whether this has been done for your work in this study.

**Answer:** Thank you. Yes, we use the dataset that has been published, and we have cited these works in our manuscript. And we have added the description of “All data generated or analyzed during this study are included in this published article.” in the part of “Availability of data and materials”.

3. Research involving human subjects (including human material or human data) that is reported in the manuscript must have been performed with the approval of an appropriate ethics committee. Research carried out on humans must be in compliance with the Helsinki Declaration (http://www.wma.net/en/30publications/10policies/b3/index.html). A statement to this effect must appear in the ‘Ethics approval and consent to participate’ section of the Declarations of the manuscript, including the name of the body which gave approval, with a reference number where appropriate.
 If the need for ethics approval were waived, then please clearly state this, including the name of the ethics committee that provided the exemption, together with the reasons for the waiver, or a reference to the relevant legislation.

**Answer:** Thank you. In our manuscript, we have used the human breast dataset to validate our method. We download this dataset from the website of <https://www.ncbi.nlm.nih.gov/geo/query/acc.cgi?acc=GSE2034>. And we have described the website information in the section of “Validation of human breast dataset”.

In addition, we have added the description of “All data generated or analyzed during this study are included in this published article.” in the part of “Availability of data and materials”.

4. For all research involving human subjects, informed consent to participate in the study should be obtained from participants (or their parent or guardian in the case of children under 16) and a statement to this effect should appear in the ‘Ethics approval and consent to participate’ section of the Declarations including whether the consent was written. When reporting on such studies, individual patient data should not be made available unless consent for publication has also been obtained.
 If the need for informed consent has been waived by an IRB or is deemed unnecessary according to national regulations, please clearly state this with details, including the name of the Board or a reference to the relevant legislation in the ‘Ethics approval and consent to participate’ section of the Declarations.

**Answer:** Thank you. In our manuscript, we have used the human breast dataset to validate our method. We download this dataset from the website of <https://www.ncbi.nlm.nih.gov/geo/query/acc.cgi?acc=GSE2034>. And we have described the website information in the section of “Validation of human breast dataset”.

In addition, we have added the description of “All data generated or analyzed during this study are included in this published article.” in the part of “Availability of data and materials”.

5. Please note that it is the responsibility of the author(s) to obtain permission from the copyright holder to reproduce figures or tables that have previously been published elsewhere. Do you require copyright permission to reproduce any of the figures? Please also be sure to include some clarification in the relevant legends as to any software used in the generation of the figures, including version number.

**Answer:** Thank you. Yes, we ensure that the figures or tables have not been published elsewhere previously. And there is not software used in the content of our figures.

6. At this stage, please upload your proofread manuscript as a single, final, clean version that does not contain any tracked changes, comments, highlights, strikethrough or text in different colours. All relevant tables/figures/additional files should also be clean versions. Figures (and additional files) should remain uploaded as separate files. Please ensure that all figures, tables and additional/supplementary files are cited within the text. Should you wish to respond to these revision requests, please include the information in the designated input box only.

**Answer:** Thank you. Yes, we have upload our proofread manuscript as a single, final, clean version that does not contain any tracked changes, comments, highlights, strikethrough or text in different colors. We ensure that all figures, tables and additional/supplementary files are cited within the text. We also include the information in the designated input box only.

Best regards,

Jianxiao Liu
